# A Comparative Evaluation of Post-operative Pain in Patients Undergoing Root Canal Treatment With Four Different Types of Sealers: A Randomized Controlled Trial

**DOI:** 10.7759/cureus.84766

**Published:** 2025-05-25

**Authors:** Sanya Sabharwal, Pravin Kumar, Vinay Kumar Chugh, Ankur Gupta, Karishma Pathak, Arun Patnana

**Affiliations:** 1 Conservative Dentistry and Endodontics, All India Institute of Medical Sciences, Jodhpur, Jodhpur, IND; 2 Orthodontics and Dentofacial Orthopedics, All India Institute of Medical Sciences, Jodhpur, Jodhpur, IND; 3 Mechanical Engineering, Indian Institute of Technology, Jodhpur, IND; 4 Pedodontics and Preventive Dentistry, All India Institute of Medical Sciences, Rajkot, Rajkot, IND

**Keywords:** ah plus, apical extrusion, bioroot rcs, nishika canal sealer bg, postoperative pain, radiopacity, root canal sealers, tubliseal

## Abstract

Objective: This study aimed to comparatively evaluate post-operative pain, unintentional apical extrusion, and radiopacity following root canal therapy using four different classes of sealers.

Material and methods: Hundred patients requiring root canal treatment in single-rooted teeth diagnosed with symptomatic irreversible pulpitis, with or without symptomatic apical periodontitis, were recruited. Pre-operative pain levels were recorded using the visual analog scale. Patients were randomized into four sealer groups (Tubli-Seal: Kerr Endodontics, California; AH Plus: Dentsply Sirona, Charlotte; BioRoot RCS: Septodont Healthcare India Pvt. Ltd., Maharashtra, India; and Nishika Canal Sealer BG: Nippon Shika Yakuhin, Shimonoseki, Japan). A single operator performed the treatment in a single visit, using the crown-down technique with Hand Protaper instruments (Dentsply Tulsa Dental, Tulsa), irrigation with 3% sodium hypochlorite (Dentpro, Mohali, India), 17% ethylenediaminetetraacetic acid (EDTA) (MD-Cleanser, EDTA Solution, Meta Biomed, Korea), and distilled water, followed by obturation using the cold lateral condensation technique. Post-operative pain levels were recorded at 24 hours, 72 hours, and seven days. The Kruskal-Wallis H test was employed to compare the pre- and post-operative pain scores across the groups.

Results: Post-operative pain, apical extrusion, and radiopacity showed no statistically significant differences among the four sealer groups (p > 0.05). However, BioRoot RCS had the lowest pain levels at 24 and 72 hours, Tubli-Seal showed the least apical extrusion, and AH Plus and BioRoot RCS exhibited higher and comparable radiopacity.

Conclusion: All the sealers perform similarly in regards to post-operative pain reduction, unintentional apical extrusion, and radiopacity with minor variations.

## Introduction

Endodontic therapy aims to remove inflamed/necrotic pulp tissue, micro-organisms, and their toxic products from root canal systems. It involves enlarging and shaping the root canal space and sealing it with an inert material to provide a hermetic barrier between the pulp space and the periapical tissues [1].

Post-operative pain represents a frequently encountered complication associated with endodontic treatment. It is defined as “discomfort experienced after the completion of root canal treatment,” affecting 25% to 40% of patients irrespective of pulpal or periradicular conditions [2,3].

Its incidence ranged between 3% and 69.3%, occurring in the first 24-48 hours following root canal treatment, though in some instances, patients may experience pain lasting three to nine days post-obturation. The pain arises from mechanical, chemical, or microbial insult to the periradicular tissues; common causes include periapical pathology, missed canals, inadequate cleaning and shaping, apical debris extrusion, failure to maintain adequate apical patency during instrumentation and extrusion of irrigants, intracanal medications, and obturating materials [4,5].

Despite the fact that the sealer is simply employed as an adjunct material in obturation, its selection is a critical decision influencing the outcome of root canal therapy [6]. They exhibit neurotoxicity to some extent, causing peripheral sensitization [2] when they contact the periradicular tissues through the apical foramen, accessory canals, or lateral canals, inducing a local inflammatory reaction, and their varying compositions significantly influence the extent of inflammation and the resulting intensity of post-operative pain [5,7].

In the present study, we used a zinc oxide eugenol-based sealer having a longstanding history of successful use; a resin-based root canal sealer regarded as the gold standard among root canal sealers because of its physicochemical properties [8]; a calcium silicate-based root canal sealer; and a bioactive glass-based root canal sealer acclaimed for their biocompatibility and tissue healing capacities [9,10].

Sealers must also possess sufficient radiopacity to differentiate them from adjacent anatomical structures for correct placement of the sealer and to help assess the quality of root canal treatment, hence monitoring the treatment's success [11].

Thus, the primary objective of this study is to comparatively evaluate the incidence of post-operative pain using four different classes of sealers (Tubli-Seal: Kerr Endodontics, California; AH Plus: Dentsply Sirona, Charlotte; BioRoot RCS: Septodont Healthcare India Pvt. Ltd., Maharashtra, India; and Nishika Canal Sealer BG: Nippon Shika Yakuhin, Shimonoseki, Japan) following root canal therapy in patients diagnosed with symptomatic irreversible pulpitis with or without symptomatic apical periodontitis. The secondary objectives are to comparatively evaluate the incidence of unintentional apical extrusion and the radiopacity of four different classes of root canal sealers. The null hypothesis stated that no significant differences would be observed among the sealer groups in these outcomes.

## Materials and methods

**Figure 1:**
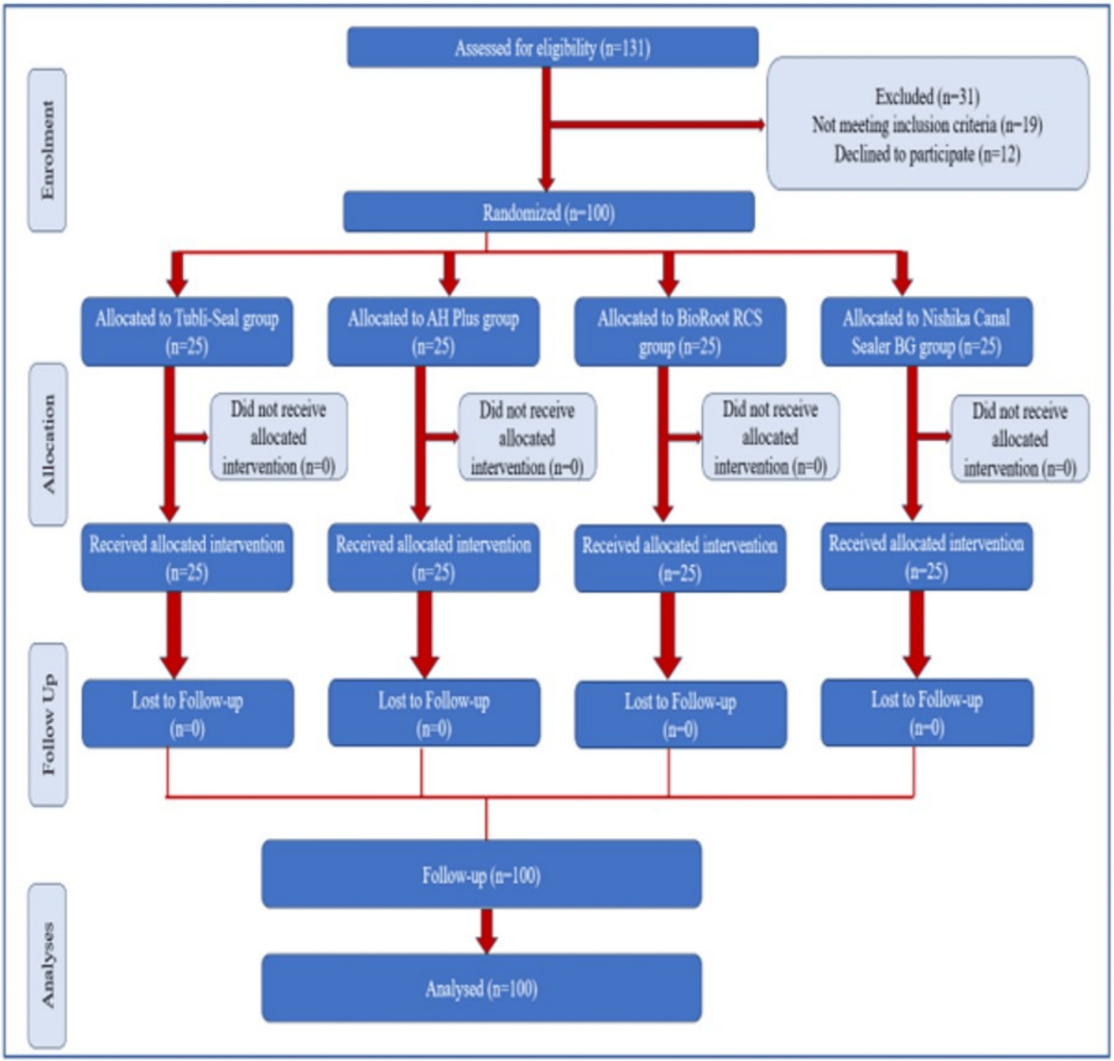
CONSORT flow diagram representing the flow of participants through each stage of a randomized trial. CONSORT: Consolidated Standards of Reporting Trials.

This study is an Experimental Parallel Randomized Clinical Trial conducted in the Department of Dentistry at the All India Institute of Medical Sciences (AIIMS), Jodhpur, which obtained ethical clearance from the Institutional Ethics Committee (Ref No.: AIIMS/IEC/2023/4464) and complied with the Helsinki Declaration of 1975. Following the Consolidated Standards of Reporting Trials (CONSORT) guidelines (Figure 1), the study was further registered with the CTRI (CTRI/2023/04/051599).

Sample size calculation

Considering the proportions in previous research by Shashirekha et al. [12], the standard deviations of the visual analog scale (VAS) scores at six and 24 hours were 32.04 and 16.35, respectively, with corresponding mean values of 35.17 and 14.07. To achieve a study power of 80% and a level of significance of 0.05 (95% confidence interval), a sample size of 80 patients (20 per group) was derived using n-Master Software (Department of Biostatistics, Christian Medical College, Vellore, India). To account for potential dropouts (attrition rate 20%) during the follow-up period, the sample size was increased to 100 patients (25 per group).

Patient enrollment

The study enrolled 100 healthy patients (American Society of Anesthesiologists (ASA) I or II) between September 2023 and September 2024, aged 18 to 65 years, with single-rooted teeth and a single apical foramen (Table 1) (Vertucci's type I & type II) diagnosed with symptomatic irreversible pulpitis, with or without symptomatic apical periodontitis. The diagnosis was supported by a clinical history of prolonged thermal sensitivity, spontaneous or referred pain, tenderness on percussion, and positive responses to the electric pulp test (Vitality Scanner Model 2006, Sybron Endo, New York) and cold sensitivity test (ROEKO Endo-Frost, Coltene/Whaledent Inc., Cuyahoga Falls). Exclusion criteria included analgesic or anti-inflammatory drug use within 12 hours before root canal obturation, pregnancy, lactation, calcified canals, periodontal diseases, root resorption, immature apices, or previously initiated root canal therapy. Patients received a bilingual written patient information sheet, and written consent was obtained.

**Table 1:** Tooth type and number of teeth included.

Tooth type	Number of teeth included
Maxillary central incisor	1
Maxillary lateral incisor	3
Maxillary second premolar	43
Mandibular central incisor	1
Mandibular first premolar	5
Mandibular second premolar	47

Patient randomization and allocation

A total of 100 patients were randomly assigned to one of four groups (n=25 per group) using a computer-generated block randomization method to ensure balanced group sizes throughout the enrollment period. The randomization sequence was generated in blocks of 20 patients, resulting in five blocks in total, with an equal allocation ratio of 1:1:1:1 within each block.

Allocation concealment was done using the sequentially numbered, opaque, sealed envelopes (SNOSE) approach. Each envelope was made of thick, non-transparent material to prevent visual identification of contents, was sequentially numbered on the outside, and contained a folded slip of paper specifying the allocated group. The envelopes were prepared by an independent researcher who was not involved in patient recruitment or treatment. At the time of enrollment, the next envelope in the sequence was opened, thereby preserving the integrity of the randomization process and minimizing selection bias.

Blinding

This study employed a double-blind design, in which both the participants and the primary investigator responsible for outcome assessment were blinded to the treatment allocation. To maintain blinding, treatment codes were concealed using identical labeling and packaging for all interventions. However, due to the procedural nature of the intervention, an independent operator who was not involved in outcome evaluation and data analysis performed the treatments. The operator was necessarily unblinded to ensure proper administration of the treatment procedure but had no interaction with participants beyond the treatment itself and no access to study outcomes or data. Prior to the commencement of the trial, the investigator and operator underwent a calibration process to ensure reliability and consistency in patient evaluation and treatment protocol, respectively. Calibration was carried out with a sample of patients who met the inclusion criteria but were not part of the study. Through this process, the operator conducted treatment repeatedly on a subset of patients under standardized conditions, and the investigator evaluated post-operative pain, unintentional apical extrusion, and radiopacity on the same subset of patients.

Treatment protocol

A comprehensive medical and dental history of each patient was obtained before commencing treatment, documenting pre-operative information, such as age, sex, tooth number, prolonged thermal sensitivity (Endo Frost: ROEKO Endo-Frost, Coltene/Whaledent Inc., Cuyahoga Falls), positive responses to electric pulp test (Vitality Scanner Model 2006, Sybron Endo, New York) and pain levels, measured using the visual analog scale (VAS). Participants were then randomly allocated into four groups by block randomization.

1. Group 1 (n=25): Tubli-Seal zinc oxide-eugenol-based sealer (Kerr Endodontics, California)

2. Group 2 (n=25): AH Plus resin-based root canal sealer (Dentsply Sirona, Charlotte)

3. Group 3 (n=25): BioRoot RCS calcium silicate-based root canal sealer (Septodont Healthcare India Pvt. Ltd., Maharashtra, India)

4. Group 4 (n=25): Nishika Canal Sealer BG (Bioactive Glass) (Nippon Shika Yakuhin, Japan)

Patients rinsed with 10 ml of 0.2% chlorhexidine gluconate solution (Calypso, Septodont, Cedex, France) for one minute before administration of local anesthesia 2% lignocaine with 1:80,000 adrenaline (Lignox-2% A, Indoco Remedies Limited, Boisar, India). Rubber dam (Hygienic, Coltene/Whaledent Inc., Cuyahoga Falls) isolation was done, and a round diamond point from Access Cavity Set (#F0001-016, Cavity Access Set, Dentsply Maillefer, Ballaigues, Switzerland) was used under air-water coolant to remove caries and create the access opening. Missing walls were restored with a composite filling material (Spectrum® Universal Microhybrid Composite Restorative, Dentsply Sirona, Germany) using Saddle Contoured Metal Matrices (Matrix Universal Kit, Tor VM, Bangalore, India). The pulp was extirpated, and the working length was determined using an electronic apex locator (CanalPro, Coltene/Whaledent Inc., Cuyahoga Falls) with a #10.02 K-file (Dentsply Maillefer, Ballaigues, Switzerland) and confirmed radiographically. Canals were prepared using Hand ProTaper Universal Files (Dentsply Tulsa Dental, Tulsa) up to a size F4 or when clean dentinal shavings were observed within the apical third along with copious irrigation and disinfection with 3% sodium hypochlorite (Dentpro, Mohali, India), 17% EDTA (MD-Cleanser, EDTA Solution, Meta Biomed, Korea) and distilled water with 30-gauge side-vented irrigation needles (PPH Cerkamed, Stalowa Wola, Poland). After drying the canals with sterile absorbent paper points (Coltene/Whaledent Inc., Cuyahoga Falls), the sealers were mixed according to the manufacturer's instructions, applied to a gutta-percha cone (Dentsply Maillefer, Ballaigues, Switzerland) and evenly distributed along the canal walls using three counterclockwise rotations. Obturation was done using the cold lateral compaction technique, a coronal composite restoration was placed, and a periapical radiograph was taken to assess obturation density and sealer extrusion. Patients were prescribed either ibuprofen 400 mg or diclofenac sodium 50 mg (if ibuprofen was contraindicated), to be taken only for acute nociceptive episodes.

Post-operative pain assessment

Post-operative pain was assessed using the visual analog scale (VAS) at 24 hours, 72 hours, and seven days post-obturation. Pain levels were classified as no pain (0-4 mm), mild pain (5-44 mm), moderate pain (45-74 mm), or severe pain (75-100 mm).

Post-operative radiographic assessment

Digital intraoral periapical radiographs were exposed by the paralleling technique on a size-1 RVG sensor (Cefla s.c., Imola, Italy) of the X-ray unit Intraskan DC (Skanray Technologies Pvt. Ltd., Bangalore, India) set at 70 kV and 8 mA with an exposure time of 3.5 seconds. Images were saved in MyRay iRys Software (MyRay, Cefla S.C., Imola, Italy), exported in JPG format to the patient’s file, and saved in the system storage.

**Figure 2:**
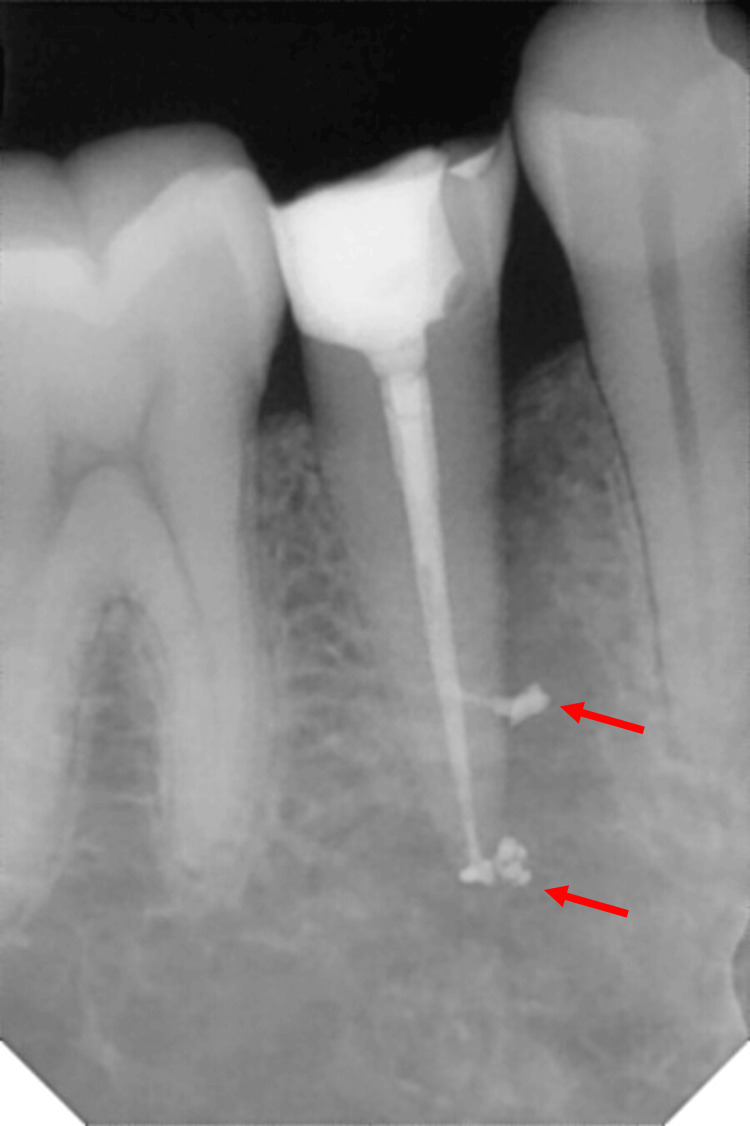
A case showing unintentional apical extrusion of sealer into the periapical region.

**Figure 3:**
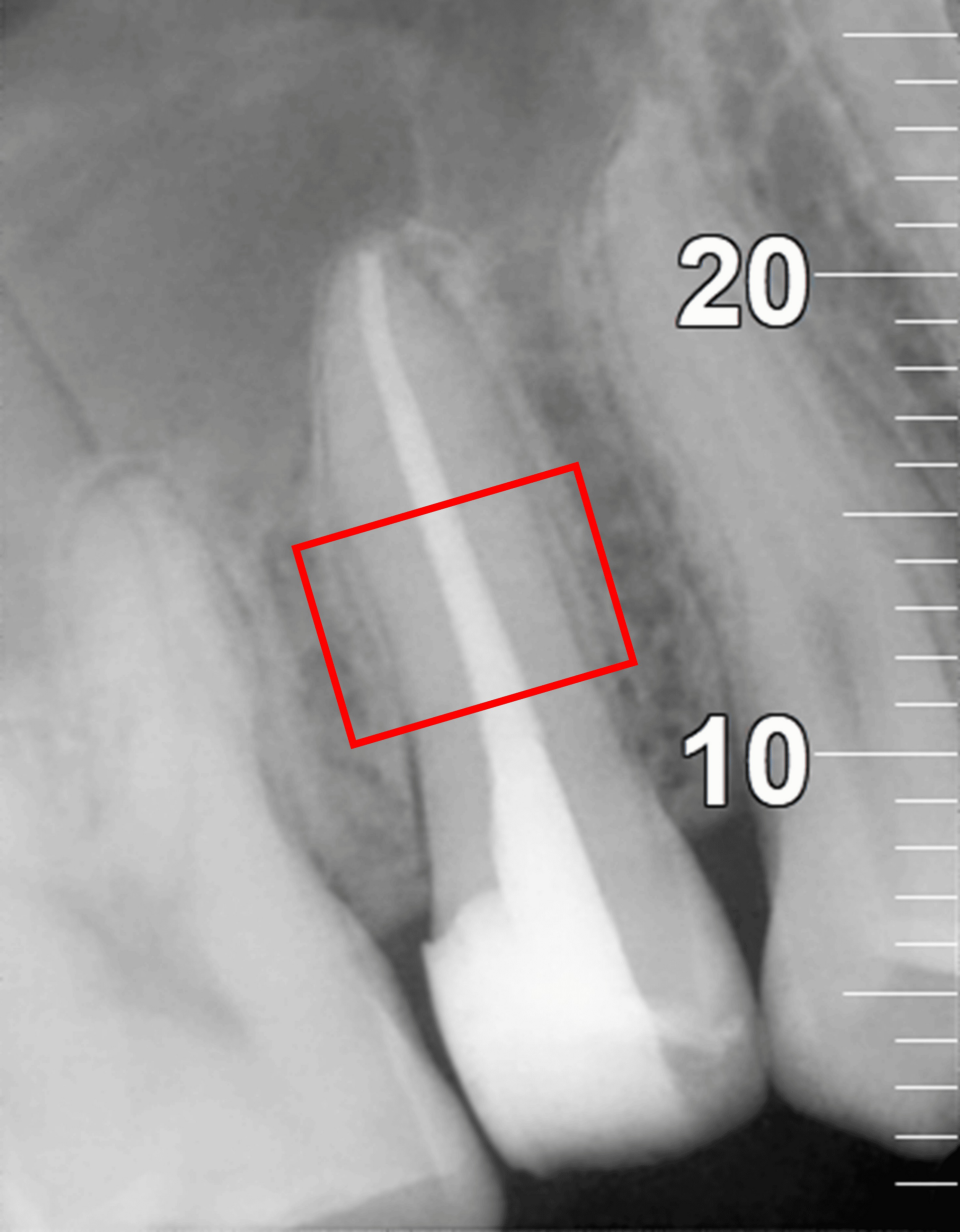
Region of interest (ROI), i.e., middle third of the root canal system marked for analysis on the radiograph to calculate the brightness percentage.

Post-operative radiographs were assessed for sealer extrusion (Figure 2), and radiopacity was calculated in terms of brightness percentage in a predetermined region of interest (ROI) corresponding to the middle third of the root canal system using ImageJ software (version 1.51, National Institute of Health, Bethesda, Fiji) (Figure 3). Each pixel in the marked region had a grayscale value ranging from 0 (black) to 255 (white). The mean grey intensity of the ROI was obtained by averaging all the grayscale values of pixels. The brightness percentage was calculated using a formula: Brightness Percentage=Mean Gray Intensity/255 × 100.

Statistical analysis

Categorical data were compared using the Chi-square test. The Shapiro-Wilk test was performed on the data to check for normality. For intergroup analysis, a one-way ANOVA test was used for normally distributed parametric data, while the Kruskal-Wallis H test was used when the data distribution was not normal. In intragroup analysis, the repeated measures ANOVA test was used for normally distributed parametric data, while the Friedman ANOVA test was used when the data were not normally distributed.

## Results

Out of 131 patients enrolled in the study between September 2023 and September 2024, 19 were excluded for not meeting the inclusion criteria, and 13 declined to participate. The remaining 100 patients who met the inclusion criteria were randomly assigned to four treatment groups (n=25).

**Figure 4:**
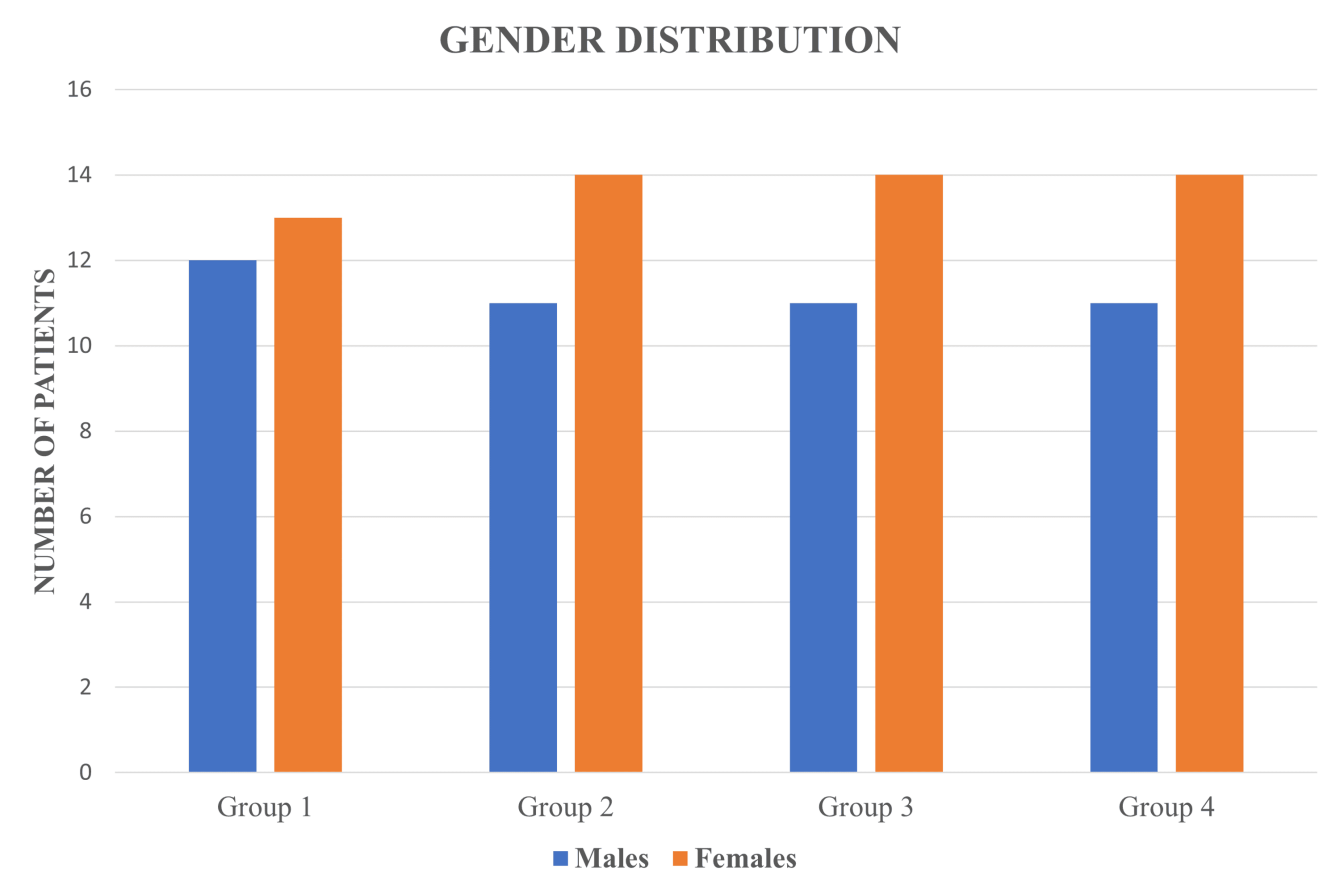
Bar chart showing gender distribution. Group 1: Tubli-Seal; Group 2: AH Plus; Group 3: BioRoot RCS; Group 4: Nishika Canal Sealer BG.

The demographic data revealed that mean ages for all groups showed slight variation, ranging from 30.160 to 34.280 years, with the age range of 18-65 years. In all four groups, there were more female patients than male patients (Figure 4). No statistically significant difference was seen between the groups in age and gender distribution (Tables 2, 3).

**Table 2:** Age distribution among the intervention groups. *One-way ANOVA test.

Variables age (in years)	Group 1: Tubli-Seal	Group 2: AH Plus	Group 3: BioRoot RCS	Group 4: Nishika Canal Sealer BG	P-value
Mean ± SD	30.560 ± 8.332	34.280 ± 10.394	30.160 ± 11.006	31.800 ± 11.321	0.493*
Median	32	32	26	30	
IQR	12	13	10	15	
Range	18-47	20-58	18-65	18-63	

**Table 3:** Gender distribution among the intervention groups. **Chi-square test.

Variables: Gender	Group 1: Tubli-Seal n (%)	Group 2: AH Plus n (%)	Group 3: BioRoot RCS n (%)	Group 4: Nishika Canal Sealer BG n (%)	P-value
Male	12 (48)	11 (44)	11 (44)	11 (44)	0.989**
Female	13 (52)	14 (56)	14 (56)	14 (56)	

**Figure 5:**
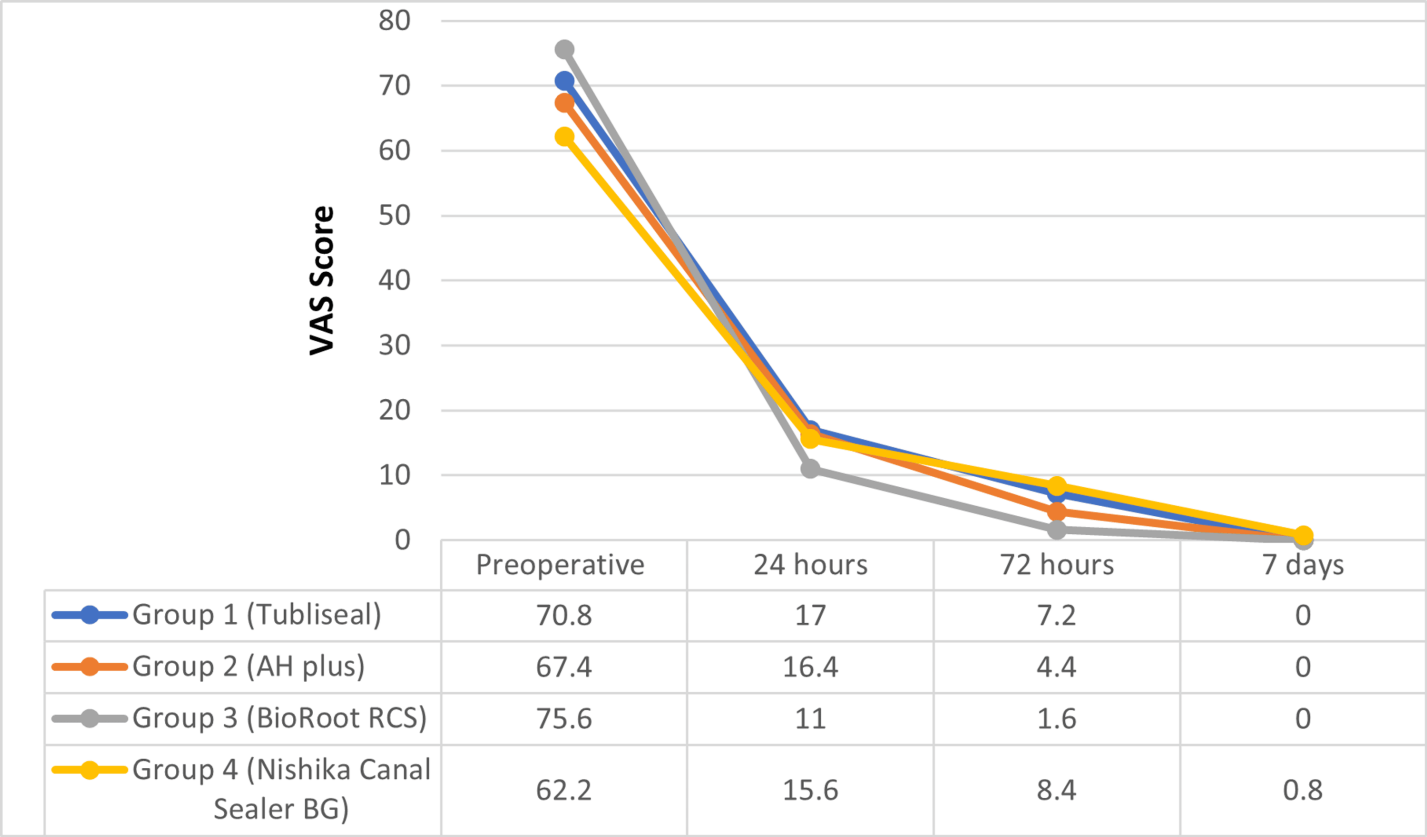
Linear chart showing the trend of mean VAS score in all four groups at different time intervals. VAS: visual analog scale.

Pre-operative and post-operative VAS scores were compared among the four groups, and the results showed no statistically significant differences at any time point (p-value > 0.05) (Figure 5, Table 4). However, group 3 demonstrated the most effective post-operative pain reduction at 24 and 72 hours. While group 4 showed the highest post-operative pain compared to the other groups at all the time intervals.

**Table 4:** VAS score, mean and standard deviation of the four groups at different time intervals. *Kruskal-Wallis H test, **Friedman ANOVA test. VAS: visual analog scale.

Variable	Group 1: Tubli-Seal	Group 2: AH Plus	Group 3: BioRoot RCS	Group 4: Nishika Canal Sealer BG	P-value (inter-group)*
Pre-operative VAS					0.442
Mean ± SD	70.800 ± 8.690	67.400 ± 22.366	75.6 ± 14.953	62.200 ± 23.544	
Median (IQR)	70 (40)	70 (30)	80(10)	70 (40)	
Range	40-100	20-100	50-100	20-100	
VAS at 24 hours					0.348
Mean ± SD	17.000 ± 20.917	16.400 ± 27.368	11.000 ± 21.699	15.600 ± 26.938	
Median (IQR)	0 (30)	0 (20)	0 (0)	0 (20)	
Range	0-60	0-90	0-70	0-80	
VAS at 72 hours					0.306
Mean ± SD	7.200 ± 17.682	4.400 ± 12.936	1.600 ± 5.538	8.400 ± 17.954	
Median (IQR)	0 (0)	0 (0)	0 (0)	0 (0)	
Range	0-80	0-50	0-20	0-60	
VAS at seven days					0.387
Mean ± SD	0	0	0	0.8 ± 4	
Median (IQR)	0 (0)	0 (0)	0 (0)	0 (0)	
Range	0	0	0	0-20	
**P-value (intra-group)	<0.001	<0.001	<0.001	<0.001	

**Figure 6:**
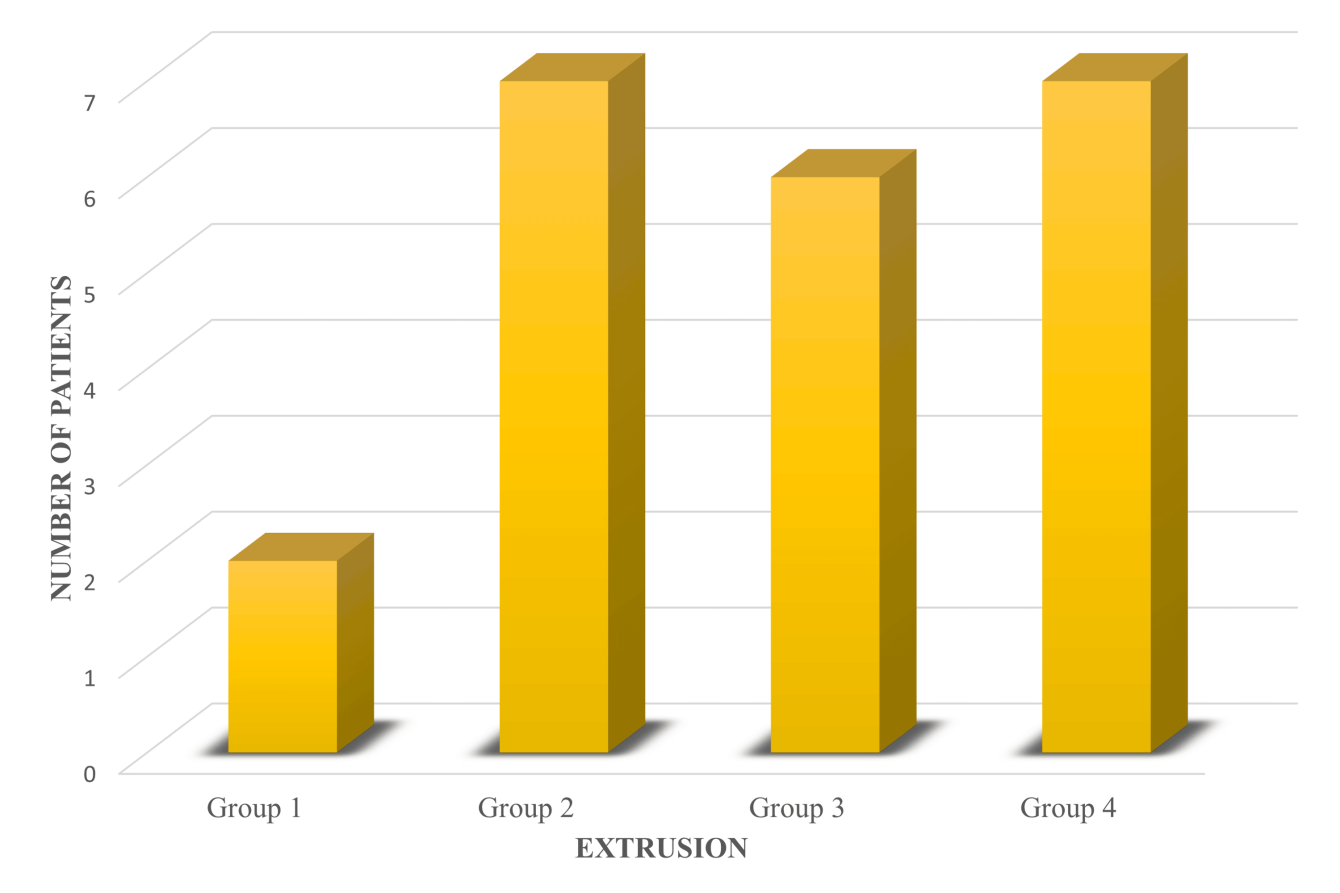
Bar chart showing the comparison of extrusion between groups. Group 1: Tubli-Seal; Group 2: AH Plus; Group 3: BioRoot RCS; and Group 4: Nishika Canal Sealer BG.

On comparing unintentional apical extrusion (Figure 6, Table 5) among the groups, no statistically significant difference was found. However, group 1 showed the lowest occurrence, while the other three groups had comparable results.

**Table 5:** Number of patients showing extrusion of sealer in the four groups. *Chi-square test.

Extrusion	Group 1: Tubli-Seal, n (%)	Group 2: AH Plus, n (%)	Group 3: BioRoot RCS, n (%)	Group 4: Nishika Canal Sealer BG, n (%)	P-value*
Present	2 (8)	7 (28)	6 (24)	7 (28)	0.265
Absent	23 (92)	18 (72)	19 (76)	18 (72)	

**Figure 7:**
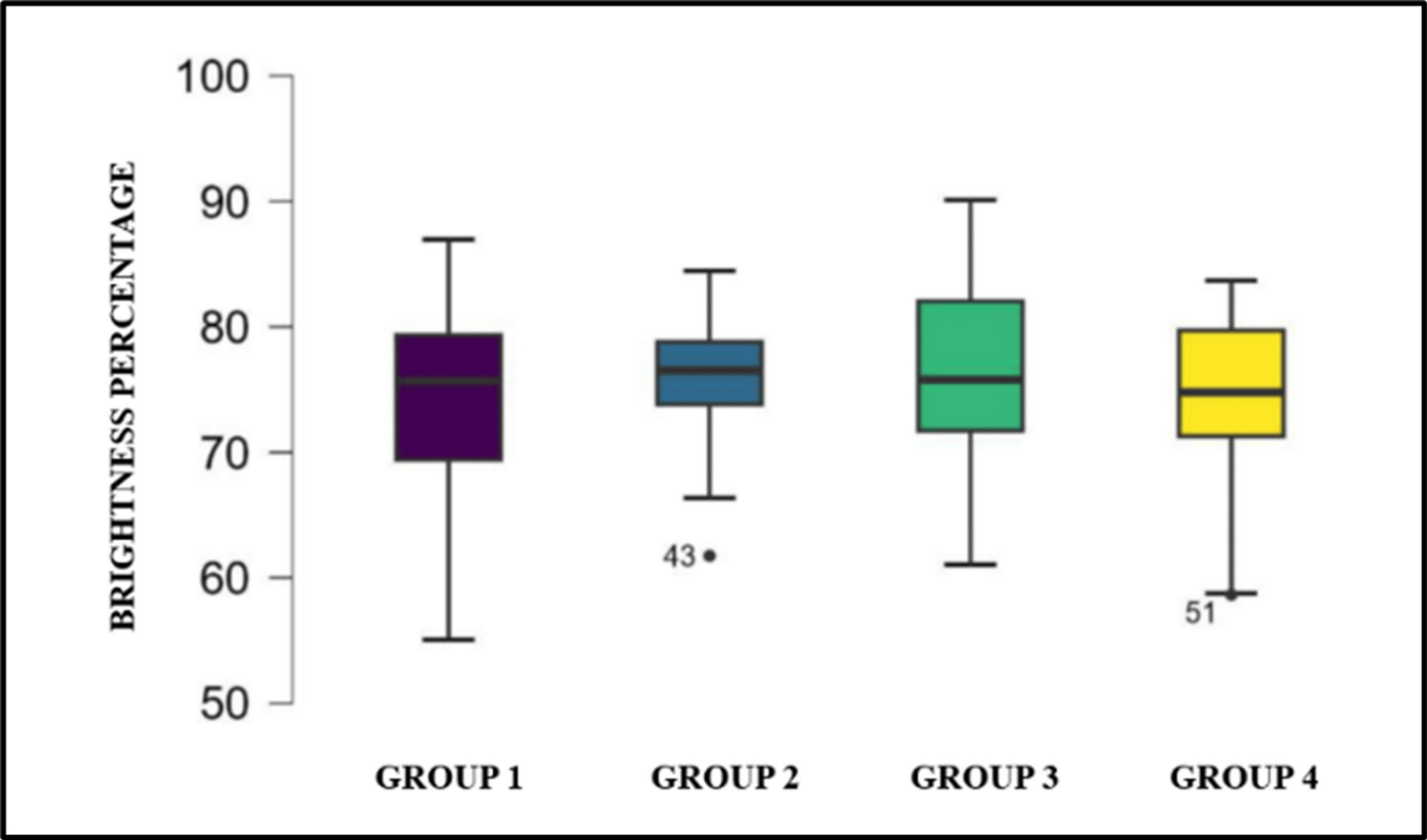
Box whisker plot showing the comparison of brightness percentage between groups. Group 1: Tubli-Seal; Group 2: AH Plus; Group 3: BioRoot RCS; and Group 4: Nishika Canal Sealer BG.

The comparison of the brightness percentage (Figure 7, Table 6) among the four groups showed no statistically significant difference. Nonetheless, the brightness percentage for group 2 and group 3 was comparable and higher than that of group 1 and group 4.

**Table 6:** Brightness percentage: mean and standard deviation, median, interquartile range, and range of the four groups. *One-way ANOVA test.

Variable	Group 1: Tubli-Seal	Group 2: AH Plus	Group 3: BioRoot RCS	Group 4: Nishika Canal Sealer BG	P-value*
Mean ± SD	74.358 ± 7.399	75.689 ± 5.292	75.828 ± 8.858	74.247 ± 7.072	0.802
Median	75.676	76.529	75.783	74.784	
IQR	9.934	4.998	10.315	8.422	
Range	55.064-86.963	61.748-84.458	61.042-90.102	58.644-83.682	

## Discussion

The incidence and severity of pain after root canal treatment are determined by patient- and treatment-related factors, often necessitating medications or emergency visits, which can be distressing for patients as well as practitioners [2,3].

The findings reported in various studies [3,7] indicated that pain following endodontic treatment typically peaks within 24-48 hours and significantly subsides by day seven.

The visual analog scale (VAS) was utilized in this study to assess the level of post-operative discomfort as it offers several advantages, including cost-effectiveness, simplicity, adaptability, and reliability as a validated measurement tool for subjective phenomena [13].

In this study, the Tubli-Seal group showed the highest mean post-operative pain score at 24 hours, followed by AH Plus, Nishika Canal Sealer BG, and BioRoot RCS. By 72 hours, Nishika Canal Sealer BG exhibited the highest post-operative pain score, while by day seven, only this group showed residual pain.

The observed results may be attributed to the direct cytotoxic effects of various components of the sealer, which leach into the periapical tissues during the setting, inducing an inflammatory reaction [5]. Reactive oxygen species (ROS) and oxidative stress are key mediators of inflammatory pain responses, with studies showing a four- to seven-fold increase in ROS in pulp cells when exposed to sealers [14].

Tubli-Seal is cytotoxic in both its unset and set states due to the presence of eugenol [5], which activates the complement system [15]. AH Plus, an epoxy resin-based sealer, releases ROS and toxic monomers like bisphenol A diglycidyl ether [14,16], causing pain, especially within the first 24 hours, and delaying periapical healing when extruded [16]. Bioceramic sealer, BioRoot RCS, releases calcium and hydroxyl ions, promoting osteogenic differentiation and tissue repair [17] by deposition of hydroxyapatite on its surface. This apatite structure provides a mechanical seal, serves as a matrix for cementum formation [17] and possesses antimicrobial properties attributed to its hydrophilicity, diffusion, and infiltration of calcium and hydroxyl ions within the dentinal tubules [18]. BioRoot RCS, in particular, exhibits low cytotoxicity and strong antibacterial activity [19], persisting for up to 30 days, contributing to the least post-operative pain.

Nishika Canal Sealer BG, a calcium silicate bioactive glass (CS-BG)-based sealer, promotes bone formation in defects by releasing ions such as calcium, phosphorus, silicon, and sodium. A silica-rich gel forms on the surface of bioactive glass, facilitating hydroxyapatite layer formation and interaction with collagen fibers, enabling BG to bond with bone. Additionally, CS-BG enhances osteoblast activity and supports bone repair and regeneration [20]. In vitro studies assessing CS-BG as a root canal sealer demonstrated its biocompatibility, with osteoblast-like cells and human periodontal ligament cells (HPDLC), showing migration and proliferation upon contact with its surface [20].

The extrusion of sealers into periapical tissues can elicit varied responses, including inflammation, foreign body encapsulation, retention of the sealer without inflammation or encapsulation, or gradual resorption [21]. Guidelines permit minimal extrusion if vital structures are unharmed [22]. Over time, macrophages phagocytose extruded sealer particles, facilitating tissue repair by promoting the proliferation of connective tissue and deposition of a cementum layer but potentially creating spaces for seepage of fluids and microbial recolonization, risking reinfection [12].

Bioceramic sealers exhibit greater flowability than resin-based sealers [23,24], though a recent study reported lower flow rates compared to AH Plus [25]. Zinc oxide eugenol-based sealers have higher viscosity and reduced flow rates than resin-based and bioceramic sealers [23]. In our study, extrusion showed no statistically significant difference: Nishika Canal Sealer BG and AH Plus had 28%, BioRoot RCS had 24%, and Tubli-Seal had the lowest at 8%.

An ideal obturation material should be radiopaque enough to differentiate it from adjacent anatomical structures like bone and teeth as well as other dental materials, including resin, amalgam, and cements. This is particularly helpful in assessing the quality of root canal treatment and detecting any voids present in the obturation [11]. Void-free obturation aids in establishing an effective seal between the pulp space and periapical tissues, preventing the ingress of periapical exudates, minimizing bacterial recolonization, and encapsulating any residual micro-organisms.

An in vitro study by Duarte et al. evaluated the radiopacity of different radiopacifiers and concluded that radiopacity decreased in the following order: bismuth oxide, zirconium oxide, calcium tungstate, barium sulfate, and zinc oxide [26]. Additionally, the potential to tailor radiopacity depends on adjusting the radiopacifier’s type, quantity, and proportions [24].

Clinical conditions differ significantly from the controlled environment of in vitro studies. In vivo, the overall radiopacity of root canal fillings is influenced by the combined densities of surrounding soft and hard tissue [27]. The composition of root canal sealers and their thickness can impact the radiopacity of the final obturation in simulated canals, highlighting the complexity of achieving accurate radiopacity measurements in clinical settings [27].

In order to calculate the radiopacity of post-operative radiographs, it was important to mark a specific region of interest (ROI) corresponding to the middle third of root canals (as a very low quantity of root canal filling material is seen in the apical third, and the radiopacity of post-endodontic restoration can affect the results in the coronal third). Each pixel in the region of interest was given a grayscale value ranging from 0 (black) to 255 (white). The brightness percentage was calculated from the mean gray values of each pixel in the marked region using ImageJ Software.

In our study, no statistically significant difference was observed in brightness percentages among groups; however, AH Plus and BioRoot RCS consistently exhibited higher brightness values due to the presence of zirconium oxide, which enhances their radiographic visibility. In contrast, Nishika Canal Sealer (with bismuth subcarbonate) and Tubli-Seal (with barium sulfate) showed lower radiopacity, reflecting the impact of their differing compositions on radiopacity levels.

To minimize iatrogenic complications, the study adopted a standardized single-visit treatment protocol in single-rooted teeth. The Hand Protaper system utilized the crown-down technique for canal preparation, reduced debris extrusion, and enhanced efficiency [28]. The cold lateral compaction technique was employed for obturation, which preserves the sealer's physical and chemical properties when compared to warm compaction techniques. The heat application can accelerate the setting reaction of AH Plus sealer by promoting the degradation of the N-H bond, leading to early polymerization. It can also significantly increase viscosity and reduce the flow of BioRoot RCS sealer [29].

Study limitations include variations in the type of tooth, its canal morphology, and the number and radiopacity of gutta-percha cones used, which influence the radiopacity outcomes more than the sealer itself, which is applied as a thin film; the subjective nature of pain perception; and limited sample size. Future randomized clinical trials with larger cohorts are necessary to validate the statistical relevance of these findings.

## Conclusions

The general trend in values obtained showed that BioRoot RCS root canal sealer had the lowest incidence of post-operative pain at 24 and 72 hours when compared to Tubli-Seal sealer, AH Plus sealer, and Nishika Canal Sealer BG. Tubli-Seal sealer showed the least apical extrusion, and AH Plus sealer and BioRoot RCS exhibited higher and comparable radiopacity.
